# School and childcare facility air quality decision-makers’ perspectives on using low-cost sensors for wildfire smoke response

**DOI:** 10.1186/s12889-023-16989-7

**Published:** 2023-11-06

**Authors:** Orly Stampfer, Stephanie Farquhar, Edmund Seto, Catherine J. Karr

**Affiliations:** 1 Department of Environmental and Occupational Health Sciences, University of Washington, 4225 Roosevelt Way NE, Seattle, WA 98105 USA; 2 Department of Health Systems and Population Health, University of Washington, 3980 15th Ave NE, Seattle, WA 98195 USA; 3 Department of Pediatrics, University of Washington, 4245 Roosevelt Way NE, Seattle, WA 98105 USA; 4 Northwest Pediatric Environmental Health Specialty Unit, 4225 Roosevelt Way NE, Seattle, WA 98105 USA

**Keywords:** Low-cost sensors, Wildfire smoke, Particulate matter, Schools, Indoor air quality, Interviews

## Abstract

**Background:**

During wildfire smoke episodes, school and childcare facility staff and those who support them rely upon air quality data to inform activity decisions. Where ambient regulatory monitor data is sparse, low-cost sensors can help inform local outdoor activity decisions, and provide indoor air quality data. However, there is no established protocol for air quality decision-makers to use sensor data for schools and childcare facilities. To develop practical, effective toolkits to guide the use of sensors in school and childcare settings, it is essential to understand the perspectives of the potential end-users of such toolkit materials.

**Methods:**

We conducted 15 semi-structured interviews with school, childcare, local health jurisdiction, air quality, and school district personnel regarding sensor use for wildfire smoke response. Interviews included sharing PM_2.5_ data collected at schools during wildfire smoke. Interviews were transcribed and transcripts were coded using a codebook developed both a priori and amended as additional themes emerged.

**Results:**

Three major themes were identified by organizing complementary codes together: (1) Low-cost sensors are useful despite data quality limitations, (2) Low-cost sensor data can inform decision-making to protect children in school and childcare settings, and (3) There are feasibility and public perception-related barriers to using low-cost sensors.

**Conclusions:**

Interview responses provided practical implications for toolkit development, including demonstrating a need for toolkits that allow a variety of sensor preferences. In addition, participants expected to have a wide range of available time for monitoring, budget for sensors, and decision-making types. Finally, interview responses revealed a need for toolkits to address sensor uses outside of activity decisions, especially assessment of ventilation and filtration.

**Supplementary Information:**

The online version contains supplementary material available at 10.1186/s12889-023-16989-7.

## Background

Wildfire smoke is increasing in frequency and severity and impacts health [1–5]. Children are particularly vulnerable to air pollution because they are still developing and their physiology results in a higher dose of air pollution compared to adults with a similar concentration of ambient exposure [6, 7]. Wildfire smoke can occur during the school year, and summertime smoke impacts childcare facilities and summer school. Decision-makers at school and childcare facilities, and those who provide guidance and recommendations to decision-makers, need air quality information to support their decisions and recommendations in the face of poor air quality resulting from wildfire smoke. Regulatory agency monitor data can be spatially sparse, especially in rural areas, and typically updates hourly at most. There is no regulatory air monitoring indoors.

Washington state (WA) provides guidance for school and childcare facility activity decisions during wildfire smoke. The 2022 guidance referenced agency monitor-derived Air Quality Index (AQI) levels for outdoor air quality information and suggested using low-cost fine particulate matter (PM_2.5_) sensors for indoor air quality information. Individuals are increasingly using low-cost sensors for more personally relevant indoor and outdoor air quality information. Personalized sensor data can be helpful for personal knowledge and behavior change, [8–12] but its utility for decision-making at a facility level has not been established to our knowledge. Further, sensors have data quality limitations and many utilize optical particle counters which require different adjustment factors for different sources of PM_2.5_ pollution [13, 14].

There is no established protocol for using sensor data to inform school and childcare facility decision-making. Our objective is to develop toolkits that provide guidance on using sensors to inform decision-making for school and childcare settings. School, childcare, local health jurisdiction (LHJ), and air quality agency personnel feedback on sensor data use and interpretation is key to effective toolkit development. We aim to better understand how low-cost sensors can support decision-making in schools and childcare facilities to respond to poor air quality, especially wildfire smoke, through the following questions: (1) How do features of data accuracy influence end-user interest in low-cost sensors as tools for decision-making? (2) What information do people hope to gain from low-cost sensors? (3) What actions do or would people take that are informed by low-cost sensor measurements?

In this study, we sought to address these questions through interviews with people who are involved in school and childcare facility air quality decision-making. These include either school or childcare facility staff, who make the decisions themselves, or air quality agencies, LHJs, or school and educational service districts, who provide suggestions or guidance to school and childcare facility staff. The two accuracy issues discussed are: (1) often low-cost sensors overestimate wildfire smoke pollution, and (2) when someone uses a handheld sensor, they are only capturing conditions at a particular moment in time, which could be different from the average exposure experience over time. These two data quality issue examples were established with PM_2.5_ data collected at four schools during wildfire smoke, and described in a separate study [15].

Participant perspectives on qualities that impact toolkit feasibility, data interpretability, and support for decision-making from the interviews will be incorporated into the development of the toolkits. The range of perspectives will be used to establish the different types of guidance that would be useful for different situations. Feasibility and the generation of interpretable data useful for decision-making will be prioritized to mitigate children’s exposures to air pollution.

## Methods

### Theoretical framework

**Fig. 1:**
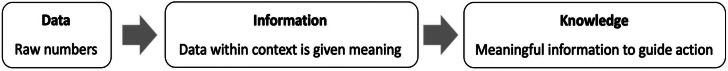
Data-Driven Decision Making theoretical framework. Not shown: implementation and iterative connection back to data collection via monitoring and evaluation of the action/change [16–18]

Qualitative interviews were useful for this study because the goal was to understand how people think about air sensor data and what impacts their decision-making or recommendations. This research was guided by a Data-Driven Decision Making theoretical framework [16, 17]. Data-driven decision making consists of *“systematic collection, analysis, examination, and interpretation of data to inform practice and policy in educational settings.”* [16] A generalized framework (Fig. 1) informed by data literacy research consists of data (raw numbers) transformed into information (data within context) and summarized into knowledge (meaningful information that can guide action) [16–18].

This theoretical framework is a good fit for this research because it is specifically relevant to collecting and using data for decision-making in educational settings. While air pollution data is not directly related to education, the context for decision-making remains in the educational setting and involves the school or childcare facility and school district, with support from local public health and air quality agencies. The processes for decision-making in the facility and school district, and the relationships between local agency staff and educational facility staff and parents are highly relevant.

The interview procedures were guided by feminist theory, including an emphasis on self-reflexivity, recognizing that the researcher cannot be separated from study design, questions, and theme identification [19–23]. These interviews included a mutual exchange of information, as participants were asked to react to air sensor data and information. Therefore, the interviews were conversational, which can help respondents think things through [19] and is a way to generate knowledge [23]. Using open-ended questions and being flexible allows for participants to describe their experiences in their own words [22] and for the research to be rooted in participants’ experiences and interpretations [23, 24].

### Interviewer positionality

The interviewer is a white, femme-presenting doctoral student with a master’s degree conducting air quality research at a large, public university in a large city. The interviewer had prior training in qualitative research methods, and experience conducting interviews and focus groups and qualitative data analysis. The interviewer recruited participants, conducted the interviews, transcribed the interviews, and was the lead researcher on developing the interview guide and conducting data analysis. The interviewer had prior research connections and/or collaborations with many of the interview participants.

### Participant recruitment

This study occurred following preliminary data analysis of air pollution data collected during wildfire smoke at four schools, described in a separate study [15]. The interviews included questions asking participants to respond to air pollution data summaries derived from the study. We sought to use a case-study approach and recruit eight specific people affiliated with the schools or school areas where we collected wildfire smoke data based on their school and agency roles, with the original intention to use snowball sampling to recruit additional participants to reach 17 people. Three people agreed to participate, two refused because their participation in the interview was out of the scope of their job responsibilities, and three did not respond.

We broadened our recruitment to 12 people at air quality agencies, LHJs, and school and educational service districts unaffiliated with the schools in the study, who either had previous research connections with the interviewer or were suggested by the WA Wildfire Smoke Impacts Advisory Group. Eight agreed to participate and four did not respond. We used snowball sampling to recruit five more people, and four participated (one did not respond). We concluded that 15 was an adequate sample size compared to our original goal of 17. In total, 15 people participated, two did not wish to participate, and eight did not respond. Respondent and non-respondent groups were similar in terms of occupation and location; the non-respondent group had slightly fewer prior connections with the interviewer.

Of the 15 participants, nine had prior research connections with the interviewer, and were aware of the interviewer’s research interests. All participants were aware of the motivations for the study. The participants from school and childcare facilities recognized air pollution as a health issue and were somewhat familiar with low-cost sensors. The participants from air quality and health agencies had previous knowledge on how air pollution harms health, air quality monitoring, and low-cost sensors. The participants who were affiliated with schools or childcare facilities were either responsible for making decisions about student activities, or they provided guidance to decision-makers. All participants had experience in their current roles of using environmental data to make decisions or provide guidance to decision-makers.

All recruitment occurred over email, and participants received and returned signed consent forms over email. The study protocol was submitted to the UW IRB and was determined to be exempt on 4/8/21 (STUDY00013077).

### Interview procedure and setting

All interviews were conducted via Zoom video conferencing between July and November 2022, and lasted from 16 to 57 min. The median interview length was 42 min. To our knowledge, all participants were in private offices or rooms at the time of the interviews without other people present. Interviews were semi-structured with an interview guide containing questions and prompts, but allowing for open dialogue and follow-up questions.

For participants from school and childcare facilities, the interview began with slides with background information on PM_2.5_ health impacts and monitoring using low-cost sensors so that participant responses could be more equally informed on relevant background to the topic. For all participants, the slides included: (1) the 2022 WA Air Quality Guide for School & Child Care Activities and explanation of how the guide motivated the study, emphasizing the indoor PM_2.5_ threshold, (2) PM_2.5_ data results demonstrating how low-cost sensor estimates differed from more expensive, “gold standard” methods of determining air concentrations of PM_2.5_, and (3) simulated PM_2.5_ data results demonstrating how estimates of PM_2.5_ derived from short momentary assessment via handheld sampling differed from average results over the wildfire smoke period (Additional File 1).

The results shown included data from outside and several indoor locations. The variation in PM_2.5_ concentrations between rooms was emphasized in the interview questions. For the school and childcare facility participants, the data shown was from their specific facility. For the other participants, the data shown was from a real facility but not one that they were affiliated with. Participants were asked to imagine that the facility was local to them when viewing the data and answering questions. The slides with data from one facility are available in Additional File 1, and the interview guide is available in Supplementary Table 1 (Additional File 2).

Prior to the interview, 14 participants completed a survey through Google Forms with short-answer and multiple-choice questions. 12 participants completed a similar post-interview survey with some identical questions. The survey included questions related to the first component of the theoretical framework, Data: raw numbers (Fig. 1). These questions focused on the feasibility of data collection with low-cost sensors during wildfire smoke, including considerations of time, cost, staff availability, and preferred sensor characteristics.

The interview guide questions were organized around the other two components of the theoretical framework: Information and Knowledge (Fig. 1). Questions focused on interpretations of low-cost sensor data and use for decision-making. Interview guide questions and prompts, organized by the theoretical framework, are available in Supplementary Table 1 (Additional File 2).

All interviews were recorded with participant permission. Repeat interviews were not conducted. The wide range of interview length is due to the difference in the number of slides shown to school and childcare facility participants vs. other participants, as well as variation in participant interest in the questions. For example, the participant with the 16-minute interview was not at all interested in using sensors indoors, so many of the questions and prompts related to using sensors indoors were irrelevant.

### Data analysis

Interviews were transcribed using Microsoft Word. Transcripts were not returned to participants for review. Transcripts were coded using Dedoose [25] qualitative software (Los Angeles, CA, USA). Deductive coding was used first; a draft codebook was developed prior to coding based on expected decisions and recommendations. New codes were added inductively as they emerged during coding. The codebook contained groups of codes organized into the following categories: Actions that people do or would take during wildfire smoke informed by low-cost sensor data, Type of decision-making, Perspectives on guidance, Types of information people hope to gain from low-cost sensor data, Barriers to using low-cost sensors, and Ways that low-cost sensors are useful. Additionally, there were several stand-alone codes: Perspectives on health impacts, Preferred data averaging time for decision-making, Mention of who would be most likely to take measurements, and Sensor preferences.

Five of the 15 transcripts were independently co-coded by the interviewer and a graduate student with qualitative research training not otherwise affiliated with the study. Co-coders were provided with background on the study, the research goals, general background on the participant roles, and a list of definitions of common abbreviations and jargon that appeared in the transcripts. The interviewer reviewed the codebook with co-coders during a meeting and answered clarifying questions.

After co-coding was complete, coding discrepancies were discussed, and mainly stemmed from lack of clarity in code definitions, differing knowledge of interview content context, and forgetting about codes due to the high number of codes. After modifying the codebook to clarify code definitions and providing additional context, co-coder agreement ranged from 81 to 93%. Agreement was calculated by the number of concurrent code applications divided by the total code applications. After co-coding, two new codes were added to track additional barriers to using low-cost sensor data and making action recommendations.

Minor themes were identified by documenting the general range of responses contained in excerpts by code. The discussion includes responses represented by a small minority of the participants, or even just one participant. Major themes were identified by organizing complementary codes together. Where themes were dominated by one category of interview participant (school/childcare, school district, LHJ, or air quality agency) this was noted. The interviewer sought to identify themes beyond those that were expected prior to the start of the study by practicing self-reflexivity and keeping an open mind as they reviewed the transcripts and organized codes into themes. Participants did not provide feedback on the findings.

## Results

The interview participants were somewhat evenly affiliated with school or childcare facilities, local health jurisdictions, air quality agencies/organizations, and school districts/educational service districts (Table 1). These workplaces were located in various Tribal Nations, rural WA counties, or urban WA counties (Table 1). One workplace was located in Arizona.

**Table 1:** Description of participants

**Place of work**	**Number of participants (% out of 15)**
School or childcare facility	3 (20%)
Local health jurisdiction	5 (33%)
Air quality agency/organization	4 (27%)
School district/Educational service district	3 (20%)
**Location of work**	**Number of different Nations or Counties**
Tribal Nation	3
Rural WA county	3
Urban WA county	2

14 people filled out the pre-interview survey that gathered information on time and budget capacity for air monitoring, and sensor preferences. 12 people filled out the post-interview survey, which was very similar. Time and budget capacity for air monitoring varied widely among respondents (Supplementary Table 2 (Additional File 3)).

Only three survey respondents said they checked indoor air quality multiple times per day during wildfire smoke, but 11 said they would want to check indoor air quality readings multiple times per day based on the 2022 WA Air Quality Guide for School & Child Care Activities. Less than half of the survey respondents thought they could use a handheld sensor to collect and document measurements from six walk-throughs of a facility. All post-interview survey respondents noted that lack of funding was a barrier in implementing air quality interventions.

The most popular responses for where people looked up outdoor air quality information were: US EPA AirNow (5 people), local clean air agency website (5), the WA Smoke Blog (4), Purple Air website (4), and WA Department of Ecology website (2). Purple Air is a low-cost (<$300) PM_2.5_ monitor that displays data on a map on a publicly accessible website.

At least two survey respondents were interested in every sensor characteristic listed. More than 60% of respondents on either the pre- or post-interview survey were interested in these sensor characteristics: stationary, handheld, connects to the internet to view real time data online, battery powered, plugs into wall outlet, option to view past data, option to apply a correction factor (used to increase accuracy of the sensor data for wildfire smoke), and able to leave outside.

There were four knowledge questions about PM_2.5_ in the survey. All respondents answered correctly on three of the questions; 11 of 14 answered correctly on the last question.

**Fig. 2:**
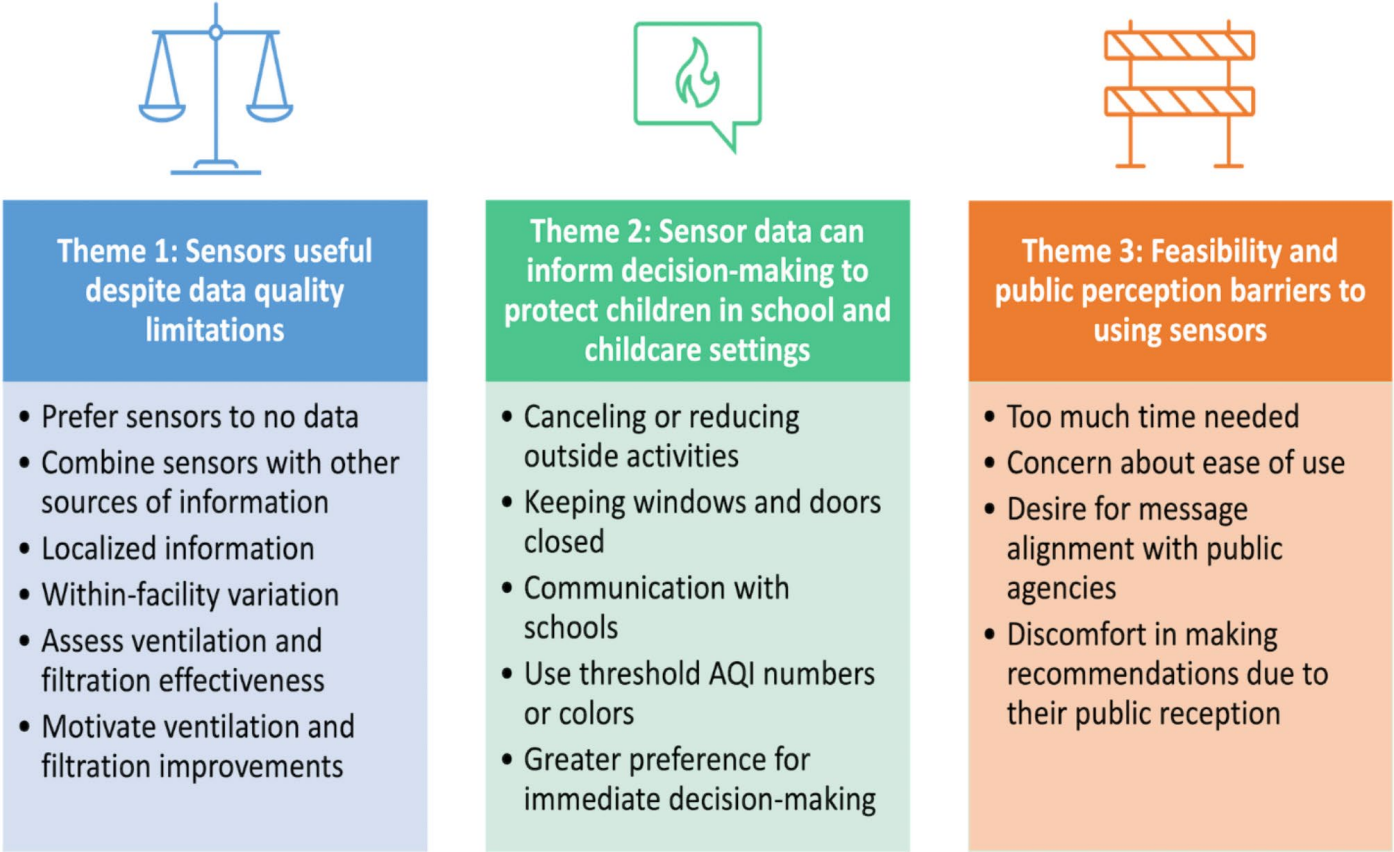
Major and minor themes from interviews

Three major themes emerged from the interviews: (1) Low-cost sensors are useful despite data quality limitations, (2) Low-cost sensor data can inform decision-making to protect children in school and childcare settings, and (3) There are feasibility and public perception related barriers to using low-cost sensors (Fig. 2).

Major themes represent general categories of perspectives on sensor use. Minor themes, listed below each major theme in Fig. 2, represent the general range of responses relevant to each theme. Generally, minor themes are associated with specific codes, while major themes are associated with groups of related codes.

### Theme 1: Low-cost sensors are useful despite data quality limitations

#### Sensors are useful despite accuracy issues

All but one of the interview participants expressed that they would use low-cost sensors despite being shown discrepancies of sensor data from more accurate data. Most participants explicitly stated that they would prefer low-cost sensors to no data. However, the participants had concerns about accuracy and expressed a desire to use the most accurate data available to them, even while agreeing that they would use the low-cost sensors. Some participants shared that at very high concentrations accuracy becomes less important because the actions would be the same at these high concentrations. Participant 6 said, referring to the 2022 WA Air Quality Guide for School & Child Care Activities:

*So if we have something where it’s – and I’m looking at the chart you had up – we know that the – it’s in one of the worst categories, we know regardless of plus or minus ten or twenty points, it still means the data we’re getting is showing that it’s unhealthy, at some level.*

Some expressed that issues with accuracy would not impact the sensors’ usefulness in identifying relative comparisons.

Because of concerns about accuracy, about half of the participants expressed that they would use the low-cost sensor data in combination with their own ideas about correction factors, comparisons to outdoor conditions, visual smoke indicators, or other sources of air quality information. Several participants noted that the sensors tend to overestimate during wildfire smoke, and shared that they preferred to overestimate than underestimate to encourage protective behavior change earlier.

#### Sensors are useful for localized information

In addition to using low-cost sensors for decision-making during wildfire smoke, which is covered in the next theme, participants shared other ways that low-cost sensor data are useful to them. About half of the participants noted that local conditions do not always reflect the AQI available for the area, and that sensors could help them get more localized information. Participant 15 said:

*Depending again on the direction of the wind, it doesn’t take much wind coming from the north, north-east, to really clear out the eastern part of our district. And then you have the southwest part of the district that is more difficult. So more [sensors] would certainly be an advantage.*

Most of the participants mentioned that handheld low-cost sensors would be useful in assessing within-school variation in air quality. Much of this interest was in identifying ventilation and filtration differences, or variation in individual behaviors from room to room, such as keeping windows and doors open.

#### Sensors are useful for assessing filtration

Most participants were interested in using sensor data to check ventilation and filtration effectiveness in general, not just how it varied throughout the school. Further, about half of the participants mentioned using sensor data to motivate ventilation and filtration improvements by sharing the data with building administrators or facilities managers.

### Theme 2: Low-cost sensor data can inform decision-making to protect children in school and childcare settings

Participants mentioned a variety of actions they would or already do take or recommend during wildfire smoke that are informed by low-cost sensor data (Table 2). Some actions were noted to be easier or more difficult due to logistics or public perception.

**Table 2:** Actions informed by low-cost sensor data

Action	Number of participants	Exemplary quote
Canceling outdoor recess or lunch	4	Participant 4: *“I don’t think I could make any sort of big asks from a school based off of Purple Air. …maybe just possibly switching recess to indoors or making some other little changes that aren’t as demanding as closing school…”*
Keeping windows and doors closed	6	Participant 10: *“If I’m trying to make a determination of, am I going to spend 3 million dollars correcting this? Then I’d probably want some research-grade meters to guide me. But if I’m talking about things like opening and closing windows, running HEPA filters, opening and closing outside air vents, that kind of stuff, I feel like those are relatively low-cost, low-risk kinds of things. So the precision and accuracy isn’t my priority at that point.”*
Reducing the rigor of indoor activities	3	Participant 3: *“With PE you could have the PE teacher try to think of a game activity that is less physically active.”*
Reducing the rigor of outdoor activities	1	Participant 2: *“I’ve encouraged coaches to restrict athletic activity, like athletic aerobic activity, you know, so maybe do drills but don’t run wind sprints.”*
Communicating with schools (relevant to non-school or childcare facility participants)	6	Participant 9: *“If one [sensor] inside is really off the charts compared to outside, I’m like ‘Ok what are they doing inside?’ And if it’s really bad outside and I see that reflected inside then I’m like ‘Ok so they got infiltration and what’s going on, what can I do to help with that?’”*
Avoiding areas of the school that have worse air quality	4	Participant 3: *“The actual logistics of moving a classroom for a day – logistically, 1) there’s not another spot to put them. We don’t have an extra classroom. I would certainly consider it, I’m just not sure logistically how that would work.”*
Canceling sports practices or games	3	Participant 11: *“If they are going to use Purple Air data, we want them to use corrected data if at all possible to make decisions on keeping kids inside and outside, and then sports in particular — that’s the real hot button topic, is when to cancel baseball or football or those kinds of things.”*

A few participants noted that although sensor data was useful for some actions, it would not be adequate for school closures decisions. Participant 12 said:

*“What I would say with Purple Air is, it is a good detection and reference data point. I think that when it comes into play for cost allocations, especially for public instruction, you need to have something a little bit more validated for the general public. Because there’s millions of dollars involved when you start closing hundreds of schools, let alone lost instruction time, confirming those decisions require generating a solid recommendation from irrefutable systems. I only point to the websites that Washington State Department of Health recommends, because they can be validated.”*

Most of the participants expressed that they used threshold AQI numbers or colors for most decisions, usually following guidance from a health or air quality agency. However, a few participants also mentioned broader, non-threshold-based decision-making.

Few participants preferred making one decision for the rest of the day’s activities based on the air quality at the beginning of the day, mainly for practical reasons, while more participants mentioned that they preferred immediate decision-making. Participant 15 (first quote) and participant 3 (second quote) offer opposing perspectives:

*I think that’s just a practical matter. It’s much more difficult to change that up midday than it is in the morning when a principal can pull their staff together and communicate with parents that are dropping off their kids…. So we tend to make those decisions early in the morning.*

*I’m a firm believer that it’s best to be outside, balanced with – we don’t want to be outside if it’s unhealthy. So I will wait until the last possible moment, so multiple times a day…. I think there was one day this year that we were inside all morning through several recesses. Our last lunch-time recess starts at 12, at 11:45 I looked at the air quality and it was below 150, I said we’re going outside.*

Several participants noted that their ability to make immediate decisions was important because of how air quality conditions can change throughout the day. Participant 10 said:

*We need better tools other than the AirNow broad predictions for regions, and what I expressed in that discussion was that every school needs a Purple Air so that they can make more immediate decisions about what kids are going to do or not do on any particular day, because we need them outside and moving as much as we can do.*

These participants also noted that the high temporal resolution of low-cost sensors was helpful for interpreting data and deciding when to open and close windows, doors, or dampers. During the interviews, we discussed how the Purple Air default display is a 10-minute averaging time, while government air quality data is typically displayed using a NowCast averaging time, which approximates to a three to 12-hour average. In response to questions about preferences for air quality data averaging time, two participants preferred the NowCast over a shorter-term average, two were unsure, and four preferred a shorter-term average.

### Theme 3: There are feasibility and public perception related barriers to using low-cost sensors

Most of the difficulty in using low-cost sensors was related to practical issues (Table 3).

**Table 3:** Logistical and time constraint-related barriers to using low-cost sensors

Barriers	Number of participants	Exemplary quote
Handheld sampling would take too much time	6	Participant 14: “*I think it’s unfeasible, to start with, just thinking about what I’ve witnessed the last week and half and being out in our facilities. And looking at our staffing, staffing models, and what’s going on with our building level administrators, they wouldn’t ever have time to do this, nor would I have the staffing for it.”*
Ease of use/user-friendliness	5	Participant 1: *“So like say I wasn’t here one day, or say I had meetings or something, could I hand that [taking handheld sensor measurements] off to somebody else to do? If I couldn’t do it.”*

Few participants (all from workplaces outside of a school or childcare facility) shared that low-cost sensor data would be difficult to use because they want to defer to regulatory agency authority for the sake of their reputation and public perception of their recommendations. Participant 14, who was the only participant who preferred no data to low-cost sensor data, said:

*“I would really rather not use data with erroneous information or questionable information, I would rather just not have it at all and continue to use quite frankly what a lion’s share of the people use, which would be [local] regional clean air’s website. Because that’s what most other people use, I can stand there and say: I’m doing what everybody else is using, the exact same tool, I didn’t have to go out on my own and use some slide-rule corrections and you start feeling a little isolated which is uncomfortable.”*

Several participants, again all from workplaces outside of a school or childcare facility, expressed discomfort and aversions to making recommendations to schools because of how they might be received by the public.

## Discussion

This study identified ways that people use or would like to use low-cost air sensors to inform decision-making in schools and childcare facilities during wildfire smoke, and identified barriers to sensor use. Overall, our interviews found a lot of interest in using sensors to guide activity decisions/recommendations and to gather information on ventilation and filtration system effectiveness. While we anticipated sensor accuracy issues to be a major barrier to their perceived utility, almost everyone expressed that they would still find sensor data useful despite data quality issues. Instead, time required to collect measurements, ease of use, and discomfort with public perception of making activity decisions/recommendations based on sensor data were the main barriers to sensor use. Time constraints were especially relevant for handheld sensor use.

### Implications for toolkit development

The survey and interview responses offered a range of perspectives on qualities that impact toolkit usefulness for gathering meaningful information to support decision-making. Responses suggested that multiple toolkits are needed to cover different options (Table 4). Additionally, responses indicated areas where further explanation and clarity are needed (Table 5).

**Table 4:** Survey and interview responses suggested that multiple toolkits are needed

Conclusions from survey and interview responses	Summary of specific responses
Include ways to use sensors that are not directly relevant to indoor activity guidance	• Assess ventilation and filtration • Make decisions about opening and closing windows and doors • Make decisions about canceling or reducing outdoor activities • Threshold for communicating with schools and childcare facilities
Include options for different: roles, amounts of time available, and decision-making type	• Wide range of suggestions of who would be taking measurements • People use both immediate and prior decision-making • Large range in time capacity to monitor per day of high air pollution (from < 15 min to > 2 h)
Have toolkit options for different types and quantities of sensors	• Responses covered range of budget for sensors (from <$100 to >$600 per facility) • Interest in every type of sensor characteristic

**Table 5:** Areas of further explanation and clarity needed, as indicated during interviews

Conclusions from survey and interview responses	Summary of specific responses
Explain reasons for different types of sampling	• Desire to check air quality multiple times a day, but logistically challenging • Interest in within-facility variation, including behavior and filtration differences • Handheld sensors are logistically difficult
Explain why sensor data may differ from agency monitor data	• Interest in combining sensor data with other sources of information • Interest in localized information • Concern about message alignment with agencies
Be clear about AQI vs. PM_2.5_, include colors, and explain sensor displays	• Confusion about AQI vs. PM_2.5_ concentration • Interest in using threshold AQI numbers and colors
Be clear about which protocols work for wildfire smoke and PM_2.5_ only	• Some interest in non-wildfire smoke air pollution • Most people interested in other types of air pollutants besides PM_2.5_

### Connections to other sensor studies

Other studies that have focused on personal behavior change rather than school or childcare facility activity decisions reflect some overlap with participant responses in this study. Multiple studies found that sensor data influences personal behavior change, including changes relevant to short-term exposures [8–11] which implies that the sensor data was useful at high temporal resolution. Sensor data was also noted as desirable by individuals in other studies because it allowed for greater spatial coverage [9, 12]  ability to check multiple times per day, [8] addressed multiple seasonal air quality issues, [8] and complemented agency monitor data. [12] The same sensor benefits were identified by interview responses in this study focused on school or childcare facility-based decision-making.

In one study of sensor use during wildfire smoke, participants used sensor data to decide whether to wear N95 respirators, whether to exercise, and when to go outside [8]. They also used sensors to identify places nearby with better air quality where they might be able to spend time [8]. This aligns well with school and childcare facility activity decisions discussed in this study; while interview participants did not discuss mask or respirator use, deciding whether to exercise and when to go outside are analogous to decisions on activity rigor and holding indoor vs. outdoor activities. Using sensors to identify places nearby with better air quality could be applicable to school athletics, if it is logistically feasible to hold practices or events in different locations. However, this type of decision was not raised in the interviews. This could be because the interviews emphasized indoor air quality-related decision-making.

In another study using personalized monitoring, participants changed behaviors in response to sensor readings, such as avoiding walking on busier streets and doing projects outside if they emitted air pollution [9]. Other studies were focused on indoor air quality, and based on sensor readings participants increased ventilation [10, 11] and reduced incense burning [10]. In these examples, sensor use helped promote air quality awareness and people taking action to protect the health of themselves and their households and workplaces.

While these types of decisions are less relevant to school and childcare facility activity decisions in response to wildfire smoke, they speak to concerns raised by interview participants about the impact of occupant behaviors. Several interviewees discussed different occupant behaviors that impact air quality, such as art classes, wood shop, and chemistry labs. More relevant to wildfire smoke, they raised concerns about opening windows and doors, and noted that sensors could be useful to raise awareness about these actions.

Interview responses also emphasized using sensors to assess building ventilation and filtration, which is not represented in these other studies of sensor use. This is likely because the other studies were focused on individual behavior changes, rather than facility-level changes. While many studies have examined school air quality, [26, 27] they have not investigated the use of low-cost sensors by school and childcare facility decision-makers in assessing their own air quality.

### Limitations and strengths

The interviewees’ proximity to the data shown varied – some participants viewed data from their own facility while most did not. This may have influenced how they answered the questions. Participants who viewed data from their own facility may have been more interested in the information and therefore more open to using sensors. However, there was strong interest in using sensors among nearly all the participants.

Participants selected for this study were interested in or knowledgeable about air quality issues, had experience using environmental data to make or guide decisions, and had experienced wildfire smoke. It is possible that the areas of further explanation needed identified by the interview responses do not adequately capture the range of explanations that would be helpful in general. For example, it may be helpful to provide explanations in the toolkits about air quality health impacts, to motivate the use of the toolkits by decision-makers who were previously unaware of air quality health concerns or had not previously experienced wildfire smoke impacts. Decision-makers at schools and childcare facilities that have not experienced much wildfire smoke or have not used environmental data for decision-making may not have the time or budget for using sensors. It may be helpful to explain the benefits of different types of sampling to help justify the use of staff time and facility budget for air monitoring.

The established connection that many of the participants had with the interviewer could be a limitation or a strength. It is possible that prior connections would lead the participants to respond in a way that they thought the interviewer wanted to hear due to social desirability bias. However, this seems unlikely to have impacted interview responses because they covered a wide range of opinions. Connection between the interviewer and the participants could be viewed as a strength as rapport was already present. This rapport likely led to more candid responses, as participants may have been less concerned about judgmental reactions from the interviewer.

This study provides new information on the ways people may use sensor data to inform decision-making in school and childcare settings, and builds on studies of how sensor data informs personal behavior change. Participants were from a range of workplaces and locations, including Tribal Nations, urban areas, and rural areas, potentially increasing the generalizability of this study in the Northwest. Having a greater understanding of how people think about using sensors for decision-making in schools and childcare facilities can improve guidance for school and childcare settings, facilitating behavior and building changes that reduce child and youth exposures to wildfire smoke.

## Conclusions

Three major themes were identified in the interview responses: (1) Low-cost sensors are useful despite data quality limitations, (2) Low-cost sensor data can inform decision-making to protect children in school and childcare settings, and (3) There are feasibility and public perception related barriers to using low-cost sensors. Interview responses also demonstrated a need for guidance that allows a variety of sensor preferences.

The interview responses revealed a strong interest in using low-cost sensor data to guide activity decisions/recommendations and to gather information on ventilation and filtration system effectiveness. This information can be directly applied to guidance for schools and childcare facilities to mitigate children’s exposure to PM_2.5_ from wildfire smoke. The perspectives on low-cost sensor use also have broader implications for decision-making and identifying air filtration needs in other types of buildings and settings for wildfire smoke as well as other high air pollution episodes.

### Electronic supplementary material

Below is the link to the electronic supplementary material.

Supplementary Material 1

Supplementary Material 2

Supplementary Material 3

## Data Availability

The interview transcripts generated and analyzed for this study are not publicly available to protect the privacy of interview participants. To request data from this study, please contact Orly Stampfer at ostamp@uw.edu.

## Abbreviations

WA: Washington state
AQI: Air Quality Index
PM_2.5_: Fine particulate matter
LHJ: Local health jurisdiction
