# Supplementary material for: School and childcare facility air quality decision-makers’ perspectives on using low-cost sensors for wildfire smoke response
Source: BMC Public Health. 2023 Nov 6;23:2167. doi: 10.1186/s12889-023-16989-7 (PMC10626666; doi:10.1186/s12889-023-16989-7)
Supplement: Supplementary file 1 — Supplementary Material 1 [file 12889_2023_16989_MOESM1_ESM.pdf]

Interview slides for participants from schools or childcare facilities started here

$PM_{2.5}$ and PM_{10}

Particulate matter (PM) comes in different sizes. The sizes that are usually monitored are PM with a diameter less than 10 micrometers (PM_{10}) or less than 2.5 micrometers ($PM_{2.5}$).

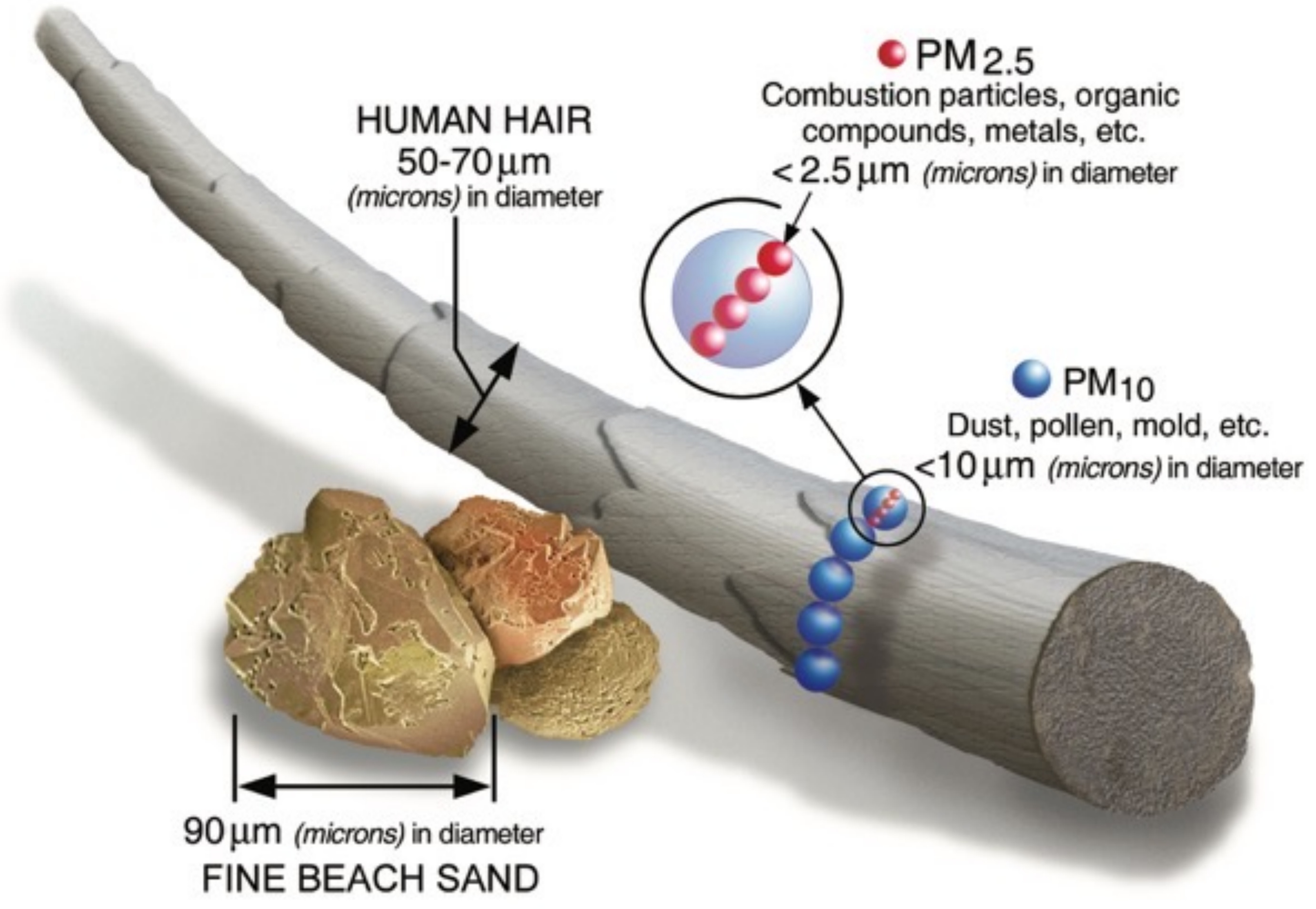

Image from the EPA: https://www.epa.gov/pm-pollution/particulate-matter-pm-basics#PM

- This slide contained background information on how PM2.5 harms cardiorespiratory health through lung inflammation, impacting the way the brain regulates the heart and circulatory system, and directly impacting the lining of blood vessels.
- It displayed a simplified version of Figure 1 from the following publication:

Miller, M. R. (2014). The role of oxidative stress in the cardiovascular actions of particulate air pollution. *Biochemical society transactions*, *42*(4), 1006-1011. https://doi.org/10.1042/BST20140090

Particle count to mass

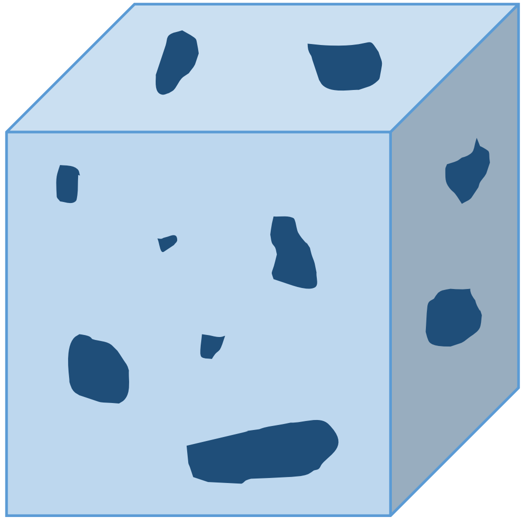

fine particles / amount of air

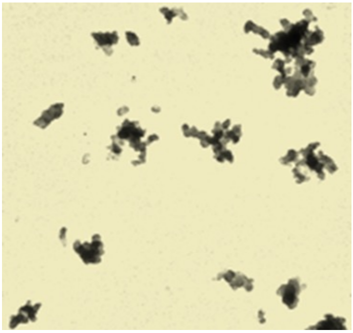

Wood smoke particles

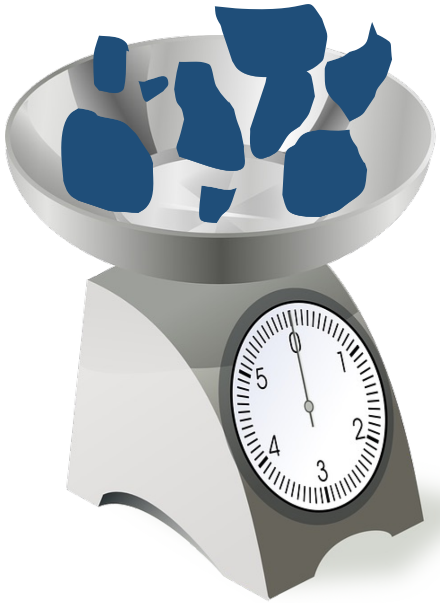

mass of fine particles / amount of air
micrograms / meter cubed
$\mu g/m^3$

- Need estimates of particle shape, volume, density
- Depends on the source and type of particles
- Challenging!

Scale image by OpenClipart-Vectors from Pixabay

Daily Air Quality Index Color	Levels of Concern	Values of Index	NowCast $PM_{2.5}$ concentration ($\mu g/m^3$)	Description of Air Quality
Green	Good	0 to 50	0-12.0	Air quality is satisfactory, and air pollution poses little or no risk.
Yellow	Moderate	51 to 100	12.1-35.4	Air quality is acceptable. However, there may be a risk for some people, particularly those who are unusually sensitive to air pollution.
Orange	Unhealthy for Sensitive Groups	101 to 150	35.5-55.4	Members of sensitive groups may experience health effects. The general public is less likely to be affected.
Red	Unhealthy	151 to 200	55.5-150.4	Some members of the general public may experience health effects; members of sensitive groups may experience more serious health effects.
Purple	Very Unhealthy	201 to 300	150.5-250.4	Health alert: The risk of health effects is increased for everyone.
Maroon	Hazardous	301 and higher	250.5-500	Health warning of emergency conditions: everyone is more likely to be affected.

Interview slides for participants from air quality or health agencies started here

Washington Air Quality Guide for School & Child Care Activities

Vehicle exhaust, woodstove emissions, industrial emissions, wildfire smoke, windblown dust, and other sources contain fine particle pollution (PM2.5) that can seriously affect children's health. The following public health recommendations to protect children from PM2.5 are designed for school activities and can be applied to child care, before/after school programs, camp, and sports programs for children (18 years and younger) by considering the duration of outdoor activities.

	**Outside Air Quality Index: PM2.5**<br>Check current and forecast air quality at enviwa.ecology.wa.gov				
	**Good**<br>(0-50)	**Moderate**<br>(51-100)	**Unhealthy for Sensitive Groups**<br>(101-150)	**Unhealthy**<br>(151-200)	**Very Unhealthy/ Hazardous**<br>(>200)
**Recess**<br>(15 minutes)	No restrictions.	Allow children with health conditions (see below*) to stay indoors.	Keep children with health conditions indoors. Keep activity levels light for these children unless indoor PM2.5 levels are below 35.5 µg/m³ (see following page).	Keep all children indoors. Keep activity levels light unless indoor PM2.5 levels are below 35.5 µg/m³.	Keep all children indoors. Keep activity levels light unless indoor air is filtered, and indoor PM2.5 levels are below 35.5 µg/m³.
**P.E.**<br>(1 hour)	No restrictions.	Allow children with health conditions to stay indoors and monitor symptoms for those who participate. Increase rest periods for these children as needed.	Keep children with health conditions indoors. Keep activities light for these children unless indoor PM2.5 levels are below 35.5 µg/m³. For others, limit to light outdoor activities. Allow any children to stay indoors if they do not want to go	Keep all children indoors. Keep activity levels light unless indoor PM2.5 levels are below 35.5 µg/m³.	Keep all children indoors. Keep activity levels light unless indoor air is filtered, and indoor PM2.5 levels are below 35.5 µg/m³.
**Athletic Events and Practices**<br>(Vigorous activity 2-3 hours)	No restrictions.	Allow children with health conditions to opt out and monitor symptoms for those who join. Increase rest periods for these children.	Cancel children's outdoor athletic events and practices or move them to an area with safer air quality, either indoors or to a different location.	Cancel children's outdoor athletic events and practices or move them to an area with safer air quality, either indoors or to a different location. Consider time spent in poor air quality during transit before relocating.	Cancel children's outdoor athletic events and practices or move them to an area with safer air quality, either indoors with filtered air or to a different location. Consider time spent in poor air quality during transit before relocating.

Health conditions include asthma and other lung disease, respiratory infection, heart disease, and diabetes. See the following page for more details about children's health, improving indoor air quality, and steps to reduce exposure.

Low-cost sensor PM2.5 measurements during school hours during wildfire smoke

	☑	?	?
Location	Low-cost sensor data corrected using accurate sampler	Uncorrected low-cost sensor data	Low-cost sensor data corrected using online map
Classroom	108	162	80
Gym	100	170	84
Cafeteria	81	129	64
Portable	82	132	65
Computer lab	N/A	136	67
Outdoors	200	260	129

Low-cost sensor PM2.5 measurements from simulated walk-through sampling during school hours during wildfire smoke

Location	Corrected low-cost sensor data	2 walk-throughs: half the time results will be between __ and __	2 walk-throughs: 95% of the time results will be between __ and __	6 walk-throughs: half the time results will be between __ and __	6 walk-throughs: 95% of the time results will be between __ and __
Classroom	108	70 and 136	46 and 186	88 and 124	66 and 150
Gym	100	73 and 122	44 and 159	83 and 112	66 and 133
Cafeteria	81	62 and 97	37 and 124	68 and 90	55 and 106
Portable	82	60 and 105	35 and 139	67 and 94	52 and 114
Computer lab	Uncorrected: 136	104 and 165	86 and 207	118 and 150	100 and 175
Outdoors	200	143 and 249	86 and 334	168 and 228	131 and 273
