# Supplementary material for: School and childcare facility air quality decision-makers’ perspectives on using low-cost sensors for wildfire smoke response
Source: BMC Public Health. 2023 Nov 6;23:2167. doi: 10.1186/s12889-023-16989-7 (PMC10626666; doi:10.1186/s12889-023-16989-7)
Supplement: Supplementary file 2 — Supplementary Material 2 [file 12889_2023_16989_MOESM2_ESM.docx]

Supplementary Table 1: Interview guide questions and prompts, organized by the theoretical framework

| Information: Data within context is given meaning | Knowledge: Meaningful information to guide action |
| --- | --- |
| What does this [low-cost sensor accuracy data] information mean to you?   - Show where numbers fall with respect to the WA Air Quality Guide for School & Child Care Activities. - Are you surprised by the differences between the rooms? Why/why not? - What questions do you have about this data? - What is more meaningful to you – the numbers or the AQI colors? | How do you use outdoor air quality information? |
| If you could only have a low-cost sensor to collect PM_2.5_ measurements, would you feel comfortable making decisions about school activities based on the low-cost sensor data?   - Does the difference of __µg/m^3^ seem important to you? Why/why not? - Would it make a difference in your decision-making? - Refer to the school activity guide - If you knew of a way to get more accurate readings (using a correction factor), would that increase your confidence in the low-cost sensor data? | How do you use indoor air quality information? |
| Are the differences between the results you get from different [sampling] methods meaningful to you?   - Does the difference of __µg/m^3^ seem important to you? Why/why not? - Would it make a difference in your decision-making? - Refer to the school activity guide | If you did feel comfortable making decisions [based on low-cost sensor data], what types of decisions would you make based on these results? How would you use these findings?   - Refer to the school activity guide - Avoid certain rooms? - Supplement air filtration in certain rooms? |
| Do you feel confident in the low-cost sensor data from the walk-throughs [handheld sampling] enough to guide your decision-making? Why/why not?   - Do you feel confident in data from two walk-throughs? Why/why not? - Do you feel confident in data from six walk-throughs? Why/why not? |  |
