# Supplementary material for: School and childcare facility air quality decision-makers’ perspectives on using low-cost sensors for wildfire smoke response
Source: BMC Public Health. 2023 Nov 6;23:2167. doi: 10.1186/s12889-023-16989-7 (PMC10626666; doi:10.1186/s12889-023-16989-7)
Supplement: Supplementary file 3 — Supplementary Material 3 [file 12889_2023_16989_MOESM3_ESM.docx]

Supplementary Table 2: Pre-interview survey responses (only the last row has a post-interview survey question).
